# An autopsy case of an adult woman with Rapid-Onset Obesity with Hypoventilation, Hypothalamic, Autonomic Dysregulation, and Neuroendocrine Tumors (ROHHAD(NET)) syndrome developing nonalcoholic steatohepatitis and hepatocellular carcinoma: A case report

**DOI:** 10.1097/MD.0000000000038383

**Published:** 2024-05-31

**Authors:** Satoru Hasuike, Yoshinori Ozono, Keisuke Uchida, Souichiro Ogawa, Hotaka Tamura, Naomi Uchiyama, Hiroshi Hatada, Yuri Komaki, Kenichi Nakamura, Hisayoshi Iwakiri, Mitsue Sueta, Kenji Nagata, Toyoki Nishimura, Misayo Matsuyama, Hirotake Sawada, Toshiyuki Oguri, Yuichiro Sato, Hiroshi Kawakami

**Affiliations:** a Faculty of Medicine, Division of Gastroenterology and Hepatology, Department of Internal Medicine, University of Miyazaki, Miyazaki, Japan; b Faculty of Medicine, Division of Pediatrics, University of Miyazaki, Miyazaki, Japan; c Faculty of Medicine, Department of Pathology, University of Miyazaki, Miyazaki, Japan.

**Keywords:** hepatocellular carcinoma, nonalcoholic steatohepatitis, ROHHAD(NET) syndrome

## Abstract

**Background:**

Nonalcoholic steatohepatitis (NASH) is an important etiology of hepatocellular carcinoma (HCC), and there is no established therapy for this syndrome. Rapid-onset obesity with hypothalamic dysfunction, hypoventilation, autonomic dysregulation, and neural crest tumor (ROHHAD(NET)) is an extremely rare syndrome considered to be life-threatening, with death occurring around 10 years of age. We present the oldest known autopsy case of this syndrome that developed HCC. This case provided important information on not only improving the course of this syndrome, but also understanding the natural history and therapeutic modalities of NASH and HCC.

**Methods:**

The patient was diagnosed with ROHHAD(NET) syndrome in childhood, and liver cirrhosis due to NASH was diagnosed at age 17. HCC was detected at age 20, and embolization and irradiation were performed. At age 21, she died from accidental acute pancreatitis and subsequent liver failure and pulmonary hemorrhage.

**Results:**

Rapid onset of obesity, hypoventilation, and hypothalamic disturbance appeared in childhood and was diagnosed as this syndrome. At age 17, liver cirrhosis due to NASH was diagnosed by liver biopsy, and at age 20, HCC was diagnosed by imaging. Transarterial chemoembolization and irradiation were performed, and the HCC was well controlled for a year.

**Conclusion:**

At age 21, she died from accidental acute pancreatitis, subsequent liver failure and pulmonary hemorrhage. Autopsy revealed that the HCC was mostly necrotized. This case was valuable not only for other ROHHAD(NET) syndrome cases, but also in improving our understanding of the natural history of NASH and HCC.

## 1. Introduction

Rapid-onset obesity with hypothalamic dysfunction, hypoventilation, and autonomic dysregulation (ROHHAD) is a rare syndrome characterized by hyperphagia and rapid-onset weight gain that starts in early childhood, and only 100 cases have been reported. This rapid and remarkable weight gain is followed by hypothalamic manifestations with neuroendocrine deficiencies, hypoventilatory breathing abnormalities, and autonomic dysregulation. The etiology of this syndrome has not been clarified, although genetic, epigenetic, and immunological theories have been suggested.^[1]^ Neural crest tumor is complicated in 4% to 50% of ROHHAD syndrome cases, and hence the acronym of the disease was amended to ROHHAD(NET) syndrome in 2008.

Natural history information about this rare syndrome is sparse because the reported mortality rates are high within a short period after diagnosis, at 50% to 60%^[2]^ usually due to hypoventilation, cardiopulmonary failure, or both.^[2–4]^

Previously, only 1 case to develop nonalcoholic steatohepatitis (NASH) and hepatocellular carcinoma (HCC), the oldest ROHHAD(NET) case, was reported.^[2]^

Nonalcoholic fatty liver disease (NAFLD) has grown from a relatively unknown disease to the most common cause of chronic liver disease worldwide. In fact, 25% of the world’s population is currently thought to have NAFLD.^[5]^ NASH is a subtype of NAFLD that can progress to cirrhosis and HCC, and NASH is already considered among the top etiologies for HCC.^[6]^

Herein, we report the published autopsy case of the second oldest (21 years old) patient with ROHHAD(NET) syndrome with liver cirrhosis due to NASH and HCC, who died from acute on chronic liver failure caused by accidental acute pancreatitis.

## 2. Case presentation

**Figure 1.**
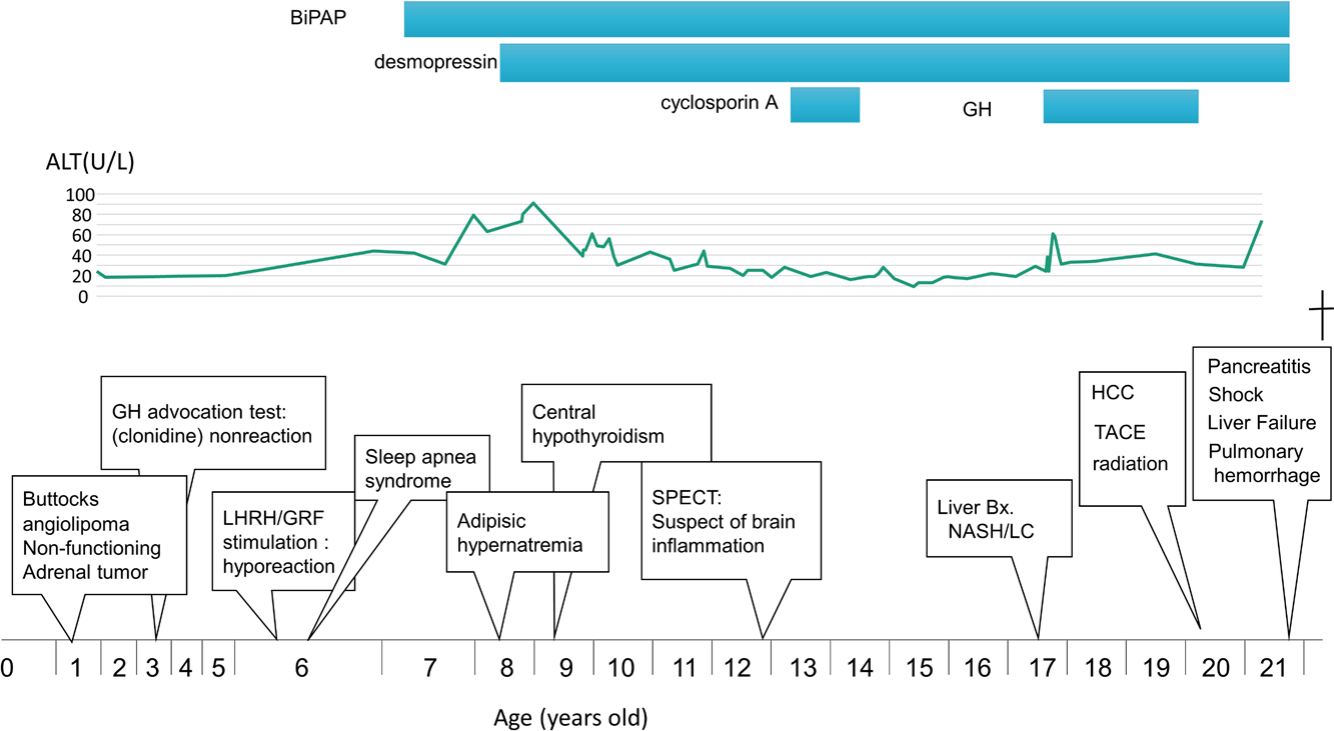
Clinical course. BiPAP = biphasic positive airway pressure, GH = growth hormone, GRF = growth-hormone-releasing factor, HCC = hepatocellular carcinoma, LC = liver cirrhosis, LHRH = luteinizing hormone-releasing hormone, NASH = nonalcoholic steatohepatitis, SPECT = single photon emission computed tomography, TACE = transarterial chemoembolization.

A 17-year-old girl was referred to us by pediatric specialists at our institution because of her liver dysfunction. Her neonatal and infancy periods were unremarkable. She had no obese family members. Aged 1, she developed a biopsy-proven angiolipoma of 40 mm in a buttock, and a 20 mm left adrenal nodule was detected accidentally (Fig. 1).

She had rapid weight gain^[7]^ and was diagnosed with growth disturbance at 3 years old. Following the results of hormonal tests, hypothalamic and pituitary disturbances were indicated, particularly in growth hormone (GH).

At 6 years old, she was diagnosed with sleep apnea syndrome, and biphasic positive airway pressure (BiPAP) was initiated from 7 years old. Additionally, at 9 years old, she was diagnosed with so-called adipsic hypernatremia.^[7]^ Her marked weight gain had not improved regardless of diet educational programs and frequent admissions for diet therapy. According to these clinical courses and findings, she was diagnosed with ROHHAD(NET) syndrome.

At 12 years old, mild brain inflammation was suspected by single photon emission computed tomography, and immunosuppressive therapy with cyclosporine was performed for a year.

Mild elevations (30–50 U/L) in alanine aminotransferase (ALT) and aspartate aminotransferase were present from 4 years old, and moderate (50–150 U/L) elevations continued from 7 to 11 years old.

At the consultation, her body height and weight were 134.1 cm and 107 kg (body mass index: 59), respectively. Hepatic cirrhosis was considered with thrombocytopenia, coagulopathy, hypoalbuminemia, and hyperammonemia (Table 1), and morphological changes and dilated collateral veins were detected by computed tomography (CT).

**Table 1:** Laboratory data.

WBC	3000 /μL	LDH	329 U/L
RBC	434 × 10^4^ /μL	ChE	309 U/L
Hb	11.2 g/dL	BUN	8.0 mg/dL
Plt	7.7 × 10^4^ /μL	Cr	0.43 mg/dL
Prothrombin time	57.9%	Glu	173 mg/dL
TP	6.72 g/dL	HbA1_C_	7.4%
Alb	3.31 g/dL	HBs-Ag	Negative
T. Bil	2.7 mg/dL	HCV-Ab	Negative
AST	45 U/L	ANA	Negative
ALT	30 U/L	AFP	19 μg/dL
ALP	434 U/L	PIVKA-II	1.4 mAU/mL
γ-GT	29 U/L	NH_3_	146 μg/dL
		Fischer ratio	1.68

γ-GT = gamma-glutamyl transpeptidase, AFP = α-fetoprotein, Alb = albumin, ALP = alkaline phosphatase, ALT = alanine aminotransferase, ANA = antinuclear antibody, AST = aspartate aminotransferase, BUN = blood urea nitrogen, ChE = cholinesterase, Cr = creatinine, Glu = glucose, Hb = hemoglobin, HbA1c = hemoglobin A1c, HBs-Ag = hepatitis B surface antigen, HCV-Ab = hepatitis C virus antibody, LDH = lactate dehydrogenase, PIVKA-II = protein induced by vitamin K absence or antagonist-II, Plt = platelets, PT = prothrombin time, RBC = red blood cells, T. Bil = total bilirubin, TP = total protein, WBC = white blood cells.

**Figure 2.**
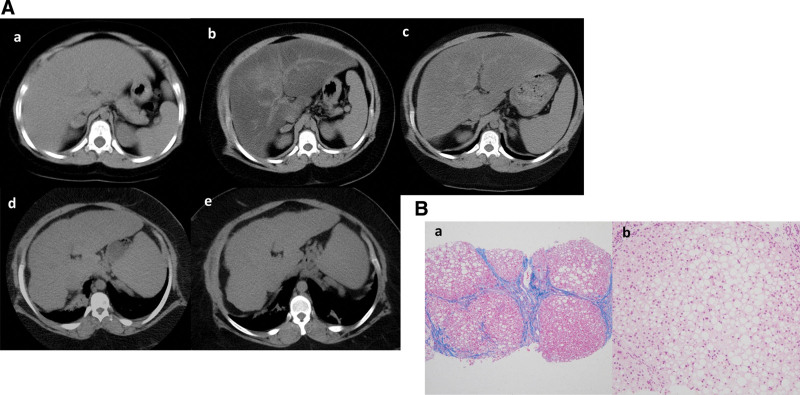
(A) Abdominal computed tomography at 4 y.o. (a), 6 y.o. (b), 8 y.o. (c), 15 yo. (d), and 17 y.o. (e). Marked hypodensity, which indicates severe fatty deposition of the liver, was observed at 6 y.o. (b) and 8 y.o. (c). The liver seemed to be atrophic and cirrhotic changes were already evident at 15 y.o. (d), and these morphological changes were more remarkable at 17 y.o. (e). The liver density at 15 and 17 y.o. was almost the same as the spleen. (B) Liver biopsy specimen at 17 y.o. (a, AZAN 4×; b, H&E 10×). Moderate steatosis, moderate lymphocytic infiltrate, and marked fibrosis were observed. According to Matteoni classification, Brunt classification, and NAS, the findings were categorized as type 4, grade 1/stage 4, and 5 points, respectively. NAS = NAFLD Activity Score, yrs old = y.o.

Liver parenchyma was retrospectively shown as isodensity at 4 years old (Fig. 2A, a), and marked hypodensity at 6 and 8 years old (Fig. 2A, b and c). The liver seemed to be atrophic and cirrhotic changes were already evident at 15 years old (Fig. 2A, d), and the change was more remarkable at this consultation. Liver density at 15 and 17 years old (Fig. 2A, d and e) was almost same as the spleen, and “burned-out NASH”^[8]^ was suspected.

Serologically (Table 1), hepatitis C antibody, hepatitis B surface antigen, and antinuclear antibody were negative. Ultrasound (US)-guided liver biopsy was performed, and liver cirrhosis with moderate inflammation and mild macrovesicular steatosis were observed histologically (Fig. 2B). From these findings, the patient was diagnosed with liver cirrhosis due to NASH and calculated as Child–Pugh B (score 8). Although GH supplementation was performed for a year, the efficacy was unclear.

**Figure 3.**
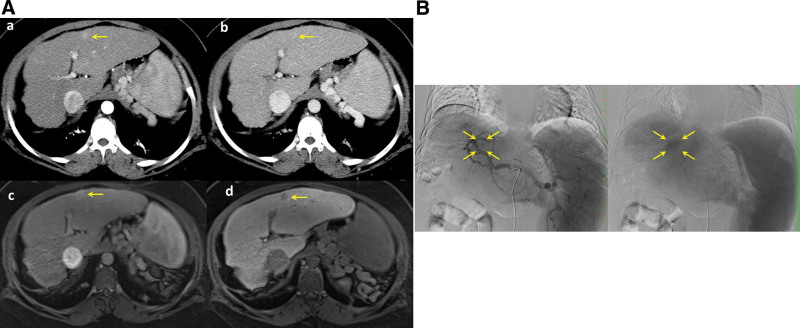
(A) Contrast enhanced abdominal CT and MRI at 19 y.o. (a, arterial and b, portal phase of CT; c, arterial and d, hepatobiliary phase of gadoxetic acid-enhanced MRI). The hypervascular nodule of 15 mm in diameter was located at the liver surface of the left lobe (arrows). (B) Hepatic arteriography from the celiac trunk. The nodule showed hypervascularity (arrows). From these findings, the tumor was diagnosed as hepatocellular carcinoma, and transarterial chemoembolization was performed. CT = computed tomography, MRI =  magnetic resonance imaging.

At 19 years old, a small liver nodule was detected by enhanced CT and magnetic resonance imaging (MRI) (Fig. 3A) on the liver surface of the left lobe. The nodule grew gradually and was diagnosed as HCC. Percutaneous radiofrequency ablation (RFA) was difficult because the nodule was located at the liver surface and US could not well detect the nodule. Surgical resection, laparoscopic RFA, and liver transplantation were considered as contraindications because general anesthesia was difficult due to her short neck, complicated asthma, and severe obesity. On angiography (Fig. 3B), the tumor was small, and the feeding arteries were unclear, and the transarterial chemoembolization (TACE) was poorly performed. Although she was transferred to another hospital with a transplantation center, transplantation was not indicated and thus irradiation therapy was performed. As a result, the tumor shrank in size, and deterioration of hepatic function was not observed. She was discharged and lived her daily life fairly well for a year.

At age 21, severe acute pancreatitis, marked electrolyte disorder, and hypovolemic shock suddenly developed, and she was admitted to an intensive care unit. Although the acute pancreatitis improved, she died from hepatic failure and pulmonary hemorrhage complicated by pulmonary edema after 20 days.

**Figure 4.**
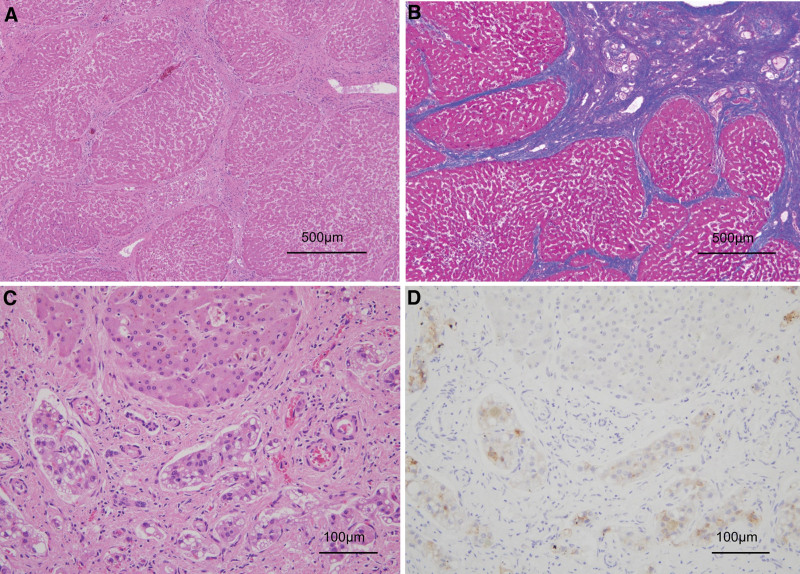
Histological findings of autopsy (liver). (a) (H&E, 4×), (b) (AZAN, 4×). The liver showed cirrhosis with fibrosis and nodular regeneration. Mild lymphoproliferation was observed, while no steatosis was seen. (c) (H&E, 20×) Findings of hepatocellular carcinoma. Cancer cells were mostly replaced by regenerative liver tissue, suggesting effects of irradiation and transarterial chemoembolization. (d) (Immunohistochemistry, 20×) Viable cancer cells were positive for glypican-3.

**Figure 5.**
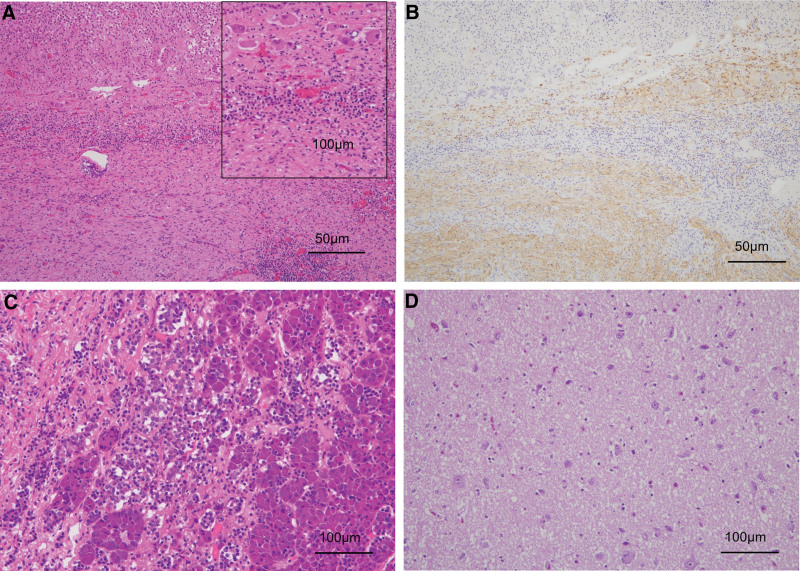
Histological findings of autopsy (others). (a) (H&E, 10×), (b) (immunohistochemistry, 10×) Histological finding of right adrenal gland. Spindle tumor cells proliferated in a fascicular pattern with ganglion cells. The tumor cells were positive for S-100 and were diagnosed as ganglioneuroma. (c) Pituitary gland (H&E, 20×) and (d) hypothalamus (H&E, 20×). No obvious pathological findings such as inflammation were observed.

Autopsy findings showed that severe liver cirrhosis was present, while steatosis in the liver was not prominent (Fig. 4A and B). The cancer cells of treated HCC were mostly necrotized and replaced by regenerated liver tissue (Fig. 4C and D). A 15 mm-sized ganglioneuroma, which characterizes this syndrome, was found in the left adrenal gland (Fig. 5A and B), and could not had been confirmed histologically antemortem. No obvious inflammation of the pituitary gland or hypothalamus was evident (Fig. 5C and D). The pancreatitis was not so prominent and seemed to be already recovered. Findings of marked pulmonary hemorrhage and edema and hepatic atrophy were also observed.

In summary, although she could have survived to adulthood despite this syndrome with poor prognosis, she suffered from NASH cirrhosis, which seems to be another complication observed in long-surviving cases, and died from acute chronic liver failure caused by accidentally complicated acute pancreatitis.

## 3. Discussion

ROHHAD(NET) syndrome is a very rare disorder associated with a high risk of mortality.^[9]^ In approximately 40% of ROHHAD patients, ganglioneuroma or ganglioneuroblastoma is observed^[10]^ and thus the acronym of the disease was amended to ROHHAD(NET) syndrome in 2008.^[10]^ The etiology of the disease remains unclear and there is still no significant genetic correlation. An autoimmune process or epigenetic disorders are currently considered as possible etiological hypotheses. Only 10 autopsy cases of this syndrome have been reported,^[11]^ and our case is believed to be the second, and hence extremely valuable, adult autopsy case. Each symptom and pathophysiological change of this syndrome seems to closely affect the development of NASH and HCC.

Sleep apnea syndrome is considered to be an independent risk factor for NAFLD because it contributes to the progression of NAFLD via oxidative stress, lipid peroxidation, inflammation, and insulin resistance.^[12]^ BiPAP treatments significantly reduce aspartate aminotransferase and ALT levels in obese patients,^[13]^ delay the progression of NAFLD, and demonstrate^[12]^ improvements in metabolic and cardiovascular functions.^[12]^ In this case, although her liver developed decompensated cirrhosis at 17 years old, the early initiation of BiPAP might have contributed to her long-term survival, preventing early death and delaying the progression of hepatic fibrosis.

GH deficiency, which was also noted in this case, is associated with NAFLD/NASH,^[14]^ and clinical application of GH and insulin-like growth factor 1 for obesity and liver cirrhosis were trialed in several pilot clinical studies.^[15,16]^ Additionally, a randomized study demonstrated that GH administration significantly improved the prognosis of patients with chronic liver failure.^[17]^ In our case, although GH supplementation was attempted for 1 year when the patient was 17 years old, the efficacy was unclear. Because over-replacement of GH may conceivably increase cancer risk,^[18]^ we had to discontinue the therapy for fear of promoting the development of buttock and adrenal tumors. Thus, the adequate scheduling and dosing of this therapy are challenging issues.

The hypothalamus has critical roles in maintaining metabolic homeostasis. Leptin signaling in the hypothalamus regulates hunger and energy expenditure. In NASH and NAFLD patients, circulating leptin levels are higher than in control subjects and levels are consistent with disease severity.^[19]^ In the current case, leptin levels when the patient was 10 years old were high and might be related to the severity of NAFLD/NASH.

Associated with the above, hypothalamic inflammation was also shown to be involved in the regulation of hepatic steatosis.^[20]^ In this case, inflammation of the hypothalamus and pituitary body had been suspected from single photon emission computed tomography data at 12 years old. Furthermore, immunosuppressive therapy was administered for a year. No active inflammation or gliosis was evident among the histological findings at autopsy, while some reported autopsy cases exhibited hypothalamic inflammation histologically. The reason was unclear, although the therapeutic effect or natural improvement due to the long survival were suspected.

According to a systematic review, the median age of death for ROHHAD(NET) syndrome is estimated at 4.6 (3–6 years).^[4]^ Another reported case with NASH and HCC was the longest living case at 27 years old; in this case, HCC had developed at age 26.^[2]^ The present case is believed to be the second-longest survival period reported at this time. This extended survival must be examined regarding the development of NASH and HCC. In the other reported ROHHAD(NET) case with HCC, RFA was successfully performed as the therapy and was well treated.

As therapy for HCC, TACE and radiation were performed in this case. According to Japanese guidelines for HCC, RFA or resection was recommended for this patient. However, the HCC of this case was located at the liver surface, and percutaneous RFA seemed to be a contraindication. Additionally, general anesthesia was intolerable because of severe obesity, asthma, and the short neck of the patient. Hence, surgical resection, laparoscopic RFA, and transplantation were also contraindications. As a result of TACE and radiation treatment, the tumor had shrunk and was controllable for a year, and the tumor was almost necrotized at autopsy.

As described above, in most ROHHAD(NET) syndrome cases, therapeutic modalities requiring general anesthesia are contraindications; therefore, some limitations for therapeutic selection may exist, and screening for HCC seemed to be more important. Jalal Eldin et al^[2]^ recommended screening for hepatic lesions using abdominal US in patients presenting with the clinical features of NAFLD as they grow older, particularly if they have signs of advanced hepatic fibrosis or cirrhosis. In our case, the HCC was detected by CT, and the US screening had not been performed. As a result, the tumor detected by CT was hardly visualized by US because of thick subcutaneal and intravisceral fat. To detect small HCCs in obese patients, CT or MRI is superior to US in general,^[21]^ and in the screening of HCC in ROHHAD(NET) syndrome, US combined with CT and/or MRI might be adequate.

The current patient died due to multiorgan failure caused by severe acute pancreatitis subsequent acute on chronic liver failure. There are no reported cases of ROHHAD(NET) developing pancreatitis. Furthermore, she had no hypertriglyceridemia or other risk factors for acute pancreatitis. Autopsy findings showed that the relationship between pancreatitis and this syndrome could not be proved.

In conclusion, we described the extremely rare autopsy case of a 21-year-old patient with ROHHAD(NET) syndrome complicated by NASH and HCC. This case was valuable not only for other ROHHAD(NET) syndrome cases, but also in improving our understanding of the natural history of NAFLD, NASH, and HCC.

## 4. Limitation

This is just a case report. Further accumulation of the cases, study and investigation with large sample size is needed.

## Acknowledgments

The authors thank H. Nikki March, PhD, from Edanz (https://jp.edanz.com/ac) for editing a draft of this manuscript.

## Author contributions

**Conceptualization:** Satoru Hasuike, Yoshinori Ozono, Yuri Komaki, Kenichi Nakamura, Mitsue Sueta, Misayo Matsuyama, Hirotake Sawada, Hiroshi Kawakami.

**Writing—original draft:** Satoru Hasuike.

**Data curation:** Keisuke Uchida, Souichiro Ogawa, Hotaka Tamura, Naomi Uchiyama, Toyoki Nishimura, Toshiyuki Oguri, Hiroshi Kawakami.

**Investigation:** Keisuke Uchida, Souichiro Ogawa, Hotaka Tamura, Hiroshi Hatada, Yuri Komaki, Kenichi Nakamura, Hiroshi Kawakami.

**Resources:** Naomi Uchiyama.

**Visualization:** Hiroshi Hatada, Toshiyuki Oguri, Yuichiro Sato, Hiroshi Kawakami.

**Supervision:** Hisayoshi Iwakiri, Mitsue Sueta, Kenji Nagata, Misayo Matsuyama, Hirotake Sawada, Toshiyuki Oguri, Yuichiro Sato, Hiroshi Kawakami.

**Writing—review & editing:** Misayo Matsuyama, Hirotake Sawada, Toshiyuki Oguri, Hiroshi Kawakami.
